# Reply: Hepatopulmonary syndrome associated with common variable immunodeficiency: Is it reasonable to investigate?

**DOI:** 10.1097/HC9.0000000000000538

**Published:** 2024-11-04

**Authors:** Neil Halliday, Siobhan O. Burns, David M. Lowe, Douglas Thorburn

**Affiliations:** 1 UCL Institute for Liver and Digestive Health, University College London, London, UK; 2 Sheila Sherlock Liver Centre, Royal Free London NHS Foundation Trust, London, UK; 3 Institute of Immunity and Transplantation, University College London, London, UK; 4 Department of Immunology, Royal Free London NHS Foundation Trust, London, UK

We thank Cassano-Santos and colleagues for their interest in our report.^1^ They raise an important question regarding the prevalence of hepatopulmonary syndrome (HPS) in patients with common variable immunodeficiency disorders (CVIDs), and portosinusoidal vascular disorders more generally. The diagnosis of HPS is based upon the triad of chronic liver disease, an elevated alveolar-arterial oxygen gradient, and the demonstration of intrapulmonary vascular dilatations or shunting, which lead to ventilation-perfusion mismatching. However, chronic lung disease itself can result in ventilation-perfusion mismatching through several mechanisms, including arterio-venous shunting in some cases.^2^ In our cohort, 78% of patients with CVID and liver involvement had either bronchiectasis or interstitial lung disease,^1^ and this high prevalence of chronic lung disease, therefore, makes the detection and diagnosis of HPS in CVID challenging.

Despite these caveats, HPS is a recognized complication in CVID and has been reported in several patients who have undergone liver transplantation.^3^^,^^4^ HPS is an independent indication for liver transplantation outside of MELD or other selection criteria in many liver transplant programs.^5^^,^^6^ Due to this and the nonstandardized selection of patients with CVID for liver transplantation, the prevalence of HPS in these reported cases is unlikely to accurately reflect that in the wider CVID population.

We did not report HPS prevalence in our study, due to variable degrees of assessment over time and the retrospective nature of our study. As noted, HPS is not diagnosed by cross-sectional imaging or pulmonary function tests but instead by dedicated investigations, including contrast-enhanced echocardiography and macro-aggregated albumin perfusion scanning. To date, we have not systematically evaluated HPS in our cohort of patients.

As liver transplantation offers survival benefits in patients with HPS^7^ and the outcome for transplantation in patients with CVID and with HPS may be favorable,^4^ we agree that it is important that evidence for HPS is sought in patients with liver disease in CVID. This is especially so in those with gas transfer defects and clinical evidence of orthodeoxia and platypnoea. Our practice now is to have a low threshold for investigation of HPS in this cohort of patients. A dedicated assessment of the prevalence, incidence, and risk factors for HPS in CVID and portosinusoidal vascular disorder would be a welcome and important study.
